# Electrically induced insulator-to-metal transition in InP-based ion-gated transistor

**DOI:** 10.1038/s41598-024-81685-4

**Published:** 2024-12-05

**Authors:** Sunao Shimizu, Hiroki Shioya, Takafumi Hatano, Kazumoto Miwa, Akira Oiwa, Shimpei Ono

**Affiliations:** 1 Fucalty of Engineering, Toyama Prefectural University, Toyama, 939-0398 Japan; 2 R3 Institute for Newly-Emerging Science Design, Osaka University, Osaka, 560-8531 Japan; 3 Department of Materials Physics, Nagoya University, Nagoya, 464-8603 Japan; 4 Materials Science Division, Central Research Institute of Electric Power Industry (CRIEPI), Kanagawa, 240-0196 Japan; 5 SANKEN, Osaka University, Osaka, 567-0047 Japan; 6 International Center for Synchrotron Radiation Innovation Smart (SRIS), Tohoku University, Miyagi, 980-8572 Japan

**Keywords:** Electronic devices, Electronic and spintronic devices

## Abstract

With the growing awareness of energy savings and consumption for a sustainable ecosystem, the concept of iontronics, that is, controlling electronic devices with ions, has become critically important. Composite devices made of ions and solid materials have been investigated for diverse applications, ranging from energy storage to power generation, memory, biomimetics, and neuromorphic devices. In these studies, three terminal transistor configurations with liquid electrolytes have often been utilized because of their simple device structures and relatively easy fabrication processes. To date, oxide semiconductors and layered materials have mainly been used as active materials. However, inorganic compound semiconductors, which have a long history of basic and applied research, hardly function as channel materials in ion-gated transistors, partly because of the Schottky barrier at the electrode interface. Herein, we show that a typical group III–V compound semiconductor, InP, is available as a high-performance channel for ion-gated transistors with an on/off current ratio of ≈ 10^5^ and a subthreshold swing as small as 93 mV/dec at room temperature. We fabricated AuGe/Ni contact electrodes via annealing to obtain the Ohmic contacts over a wide temperature range. The electrical resistance of InP was drastically decreased by the ionic liquid gating, which led to an electrically induced insulator-to-metal transition. Bulk compound semiconductors are well characterized and have relatively high carrier mobilities; thus, devices combined with electrolytes should prompt the development of iontronics research for novel device functionalities.

**Supplementary Information:**

The online version contains supplementary material available at 10.1038/s41598-024-81685-4.

## Introduction

Recent progress in iontronics, in which ions play an important role in the device architecture, has attracted significant attention from various research areas^1–4^. For example, along with conventional Li-ion batteries, all-solid-state batteries are expected to become key devices for achieving sustainable systems for human activities. In addition, in recent studies on unused energy, energy harvesting from waste heat via the ionic thermoelectric effect^5–9^ and from mechanical vibration with polarized ions^10–13^ have been studied as possible small power sources in self-powered systems. Nano scale interfaces between ions and electronic materials are ideal for the exploration of novel device functionalities.

In the study of iontronic devices, ion-gated field-effect transistors (FETs) are recognized as important research platforms^4,14,15^. This is because the physical and chemical properties of channel materials can be modified by the alignment of ions on the surface, which is controlled by an external voltage. For example, various electronic phase transitions have been realized in functional materials with FET structures^4,15^ by accumulating high-density charge carriers at the solid–liquid interface. Moreover, beyond the typical scheme of electrostatic FETs, ion intercalations^16–18^, redox reactions^15^, and neuromorphic synaptic devices^19,20^ have been demonstrated. On the other hand, however, the available channel materials for ion-gated FET configurations have been limited mostly to oxides, organic semiconductors, and two-dimensional layered materials; thus, expanding the range of these materials is crucial to accelerating the development of basic ionotronics research.

One of the target materials for the transistor channels is group III-V compound semiconductors, which have played the central roles in modern high-speed circuits by utilizing modulation-doped FET structures^21^. These compound semiconductors have moderate band gaps ranging from 0.3 eV to 1.5 eV and relatively high electron mobilities, forming sharp hetero interfaces to construct defect-free two-dimensional electron gases^21^. As a result, a high-speed switching operation, low energy consumption, and a large on/off ratio of the drain-source current *I*_D_ are realized. Thus, the application of bulk compound semiconductors to the channel materials of ion-gated FETs is utmost importance for the pursuit of high-performance iontornic devices.

The fabrication of ion-gated FETs based on III-V semiconductors has been difficult, partly because of the high contact resistance at the interface between the electrodes and channels. This problem is typically encountered when fabricating devices based on insulating materials with low Fermi levels. For example, in the case of oxide insulators such as SrTiO_3_ and KTaO_3_, Ar ion bombardment was performed to etch the underlayer of the electrodes^22,23^ and selectively remove oxygen ions. This oxygen deficiency induced thin conductive layers, which resulted in the Ohmic contact at the electrode interfaces^24–26^. As for the compound semiconductors, it was reported that the contact resistance at room temperature was reduced by employing AuGe/Ni electrodes with high-temperature annealing treatments^27–31^. This suggests that achieving low contact resistance in ion-gated devices based on III-V semiconductors over a wide temperature range can be done by finding an appropriate annealing condition through a systematic investigation of the annealing process.

**Fig. 1:**
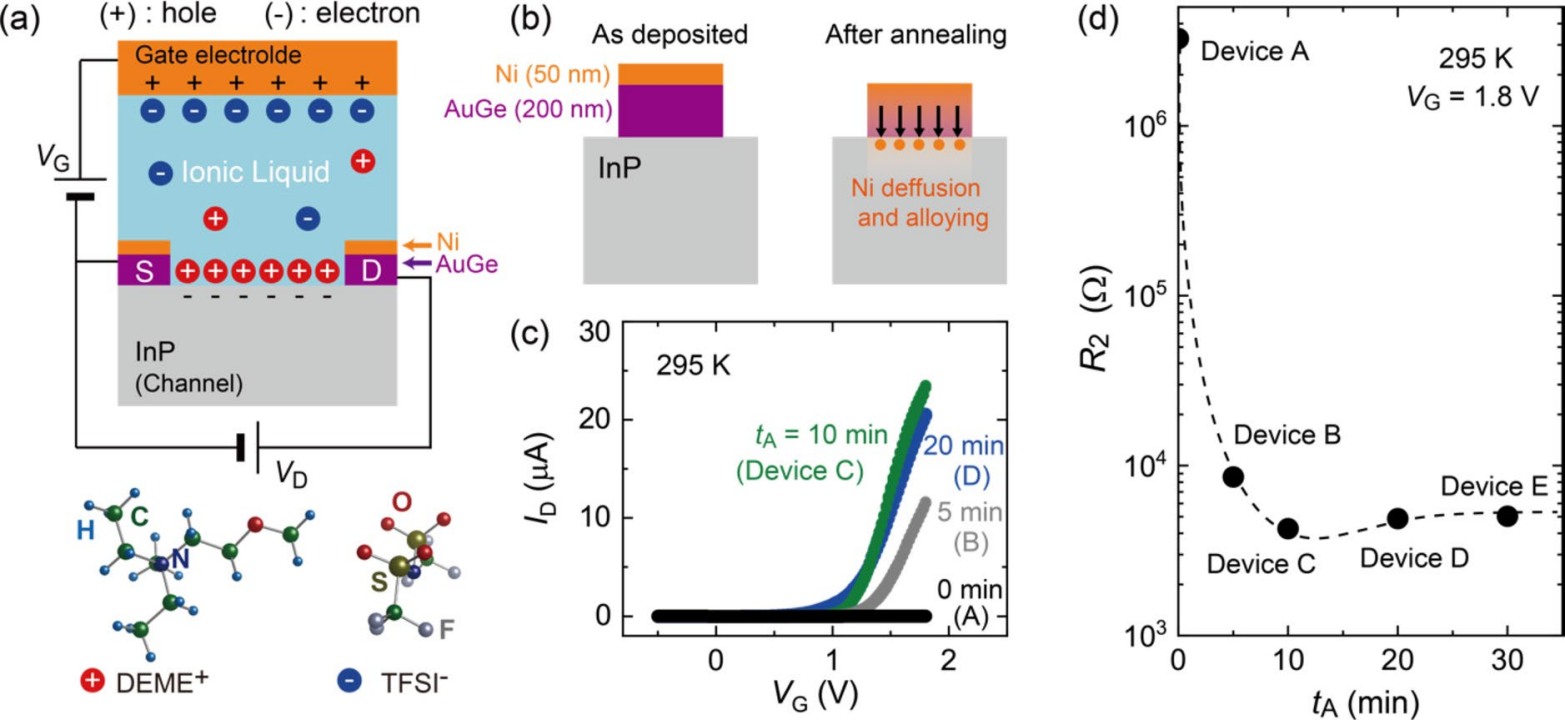
Structure of ion-gated InP device and annealing effect on contact electrodes. (a) Schematic device structure of ion-gated InP with electric double layers. When a positive gate voltage *V*_G_ is applied to the gate electrode, the cations (DEME^+^) and anions (FTSI^−^) are aligned on the channel and the gate electrode, respectively, to form the electric double layers. Here, S and D represents the source and drain electrodes, respectively. See the “Methods” for details of the device fabrication. (b) Annealing effect on contact electrodes. After annealing, Ni in the topmost layer diffuses toward the InP surface. (c) Typical transfer characteristics (drain-source current *I*_D_ versus gate voltage *V*_G_) at 295 K for different annealing times *t*_A_. The annealing temperature was 420 °C for all the measurements. The values of *I*_D_ increases with increasing *V*_G_, showing that the electron carriers are doped under positive *V*_G_ for annealed devices. The drain voltage *V*_D_ was 0.1 V. (d) Two terminal resistance *R*_2_ as a function of *t*_A_. The values of *R*_2_ at 295 K were evaluated as the resistance between the drain and source electrodes.

In this study, we present the fabrication of ion-gated FETs based on semi-insulating InP and gate control of the electrical transport properties (see the “Methods” for details of the device fabrication process and measurements). A small amount of ionic liquid was applied to the surface of an InP single crystal to form an electric double layer transistor structure, as shown schematically in Fig. 1a. AuGe/Ni electrodes with a thickness of approximately 140/35 nm were deposited by resistance heating and electron beam evaporations and subjected to a high-temperature annealing to obtain a good Ohmic contact. We systematically investigated the annealing time *t*_A_ dependences of the transfer curves (the change in *I*_D_ as a function of the gate voltage *V*_G_) and of the electrical transport at temperatures as low as 10 K. Finally, it is revealed that metallic transport was induced in the initially insulating InP by an ionic-liquid gating, which provides a further research arena for the development of iontronics using high-carrier-mobility materials.

## Results and discussion

Details of the device structure and the concept behind its fabrication are summarized in Fig. 1. Figure 1a schematically shows the device structure of the ion-gated InP, where the ionic liquid between the gate electrode and channel acts as a gate dielectric. When *V*_G_ is applied to the gate electrode, electric double layers are formed on the surface of InP and the gate electrode, inducing a strong electric field at the interfaces. The drain and source electrodes were fabricated by depositing AuGe and Ni, respectively, as shown in Fig. 1b. According to previous reports^27–31^, the contact resistance of n-type InP can be reduced using AuGe/Ni electrodes via additional annealing procedures. Ni in the topmost layer diffuses toward the InP surface through AuGe during annealing. Consequently, an intermediate semiconductor layer, possibly an alloy of Ni and P, is formed to suppress the contact resistance^30^. Figure 1c shows the transfer characteristics at 295 K for the ion-gated InP devices A (*t*_A_ = 0 min), B (*t*_A_ = 5 min), C (*t*_A_ = 10 min), and D (*t*_A_ = 20 min). The annealing temperature was set at 420 °C throughout the present study based on the discussions on making an Ohmic contact in previous studies^27–31^. When *t*_A_ was 0 min, *I*_D_ did not increase under positive *V*_G_. In contrast, *I*_D_ showed an upturn at approximately *V*_G_ = 1.0 V and increased further with increasing *V*_G_ in the annealed devices. Here, both the forward and reverse scans were plotted and mostly overlapped. This shows that the charge carriers were induced in a reversible manner against sweeping *V*_G_. Moreover, the negligible hysteresis suggests that the electrochemical reaction was unlikely. Figure 1d shows the room-temperature values of the two-terminal resistances *R*_2_ of devices A, B, C, D, and E (*t*_A_ = 30 min) for *V*_G_ = 1.8 V. The values of *R*_2_ consist of the contact resistance and channel resistance; therefore, a comparison of *R*_2_ can be a good measure for qualitatively evaluating the modulation of the contact resistance. With increasing *t*_A_, *R*_2_ significantly decreased from 3 MW to 4 kW for several minutes of annealing. This strongly suggests that the contact resistance was suppressed owing to the formation of an Ohmic contact at the interface between electrode and InP. Notably, the typical *t*_A_ value needed to obtain Ohmic electrodes in InP only ranged from several seconds to minutes in previous reports^27–30^. These recipes seem reasonable because *R*_2_ at 295 K hardly varied for longer *t*_A_, as shown in Fig. 1d.

**Fig. 2:**
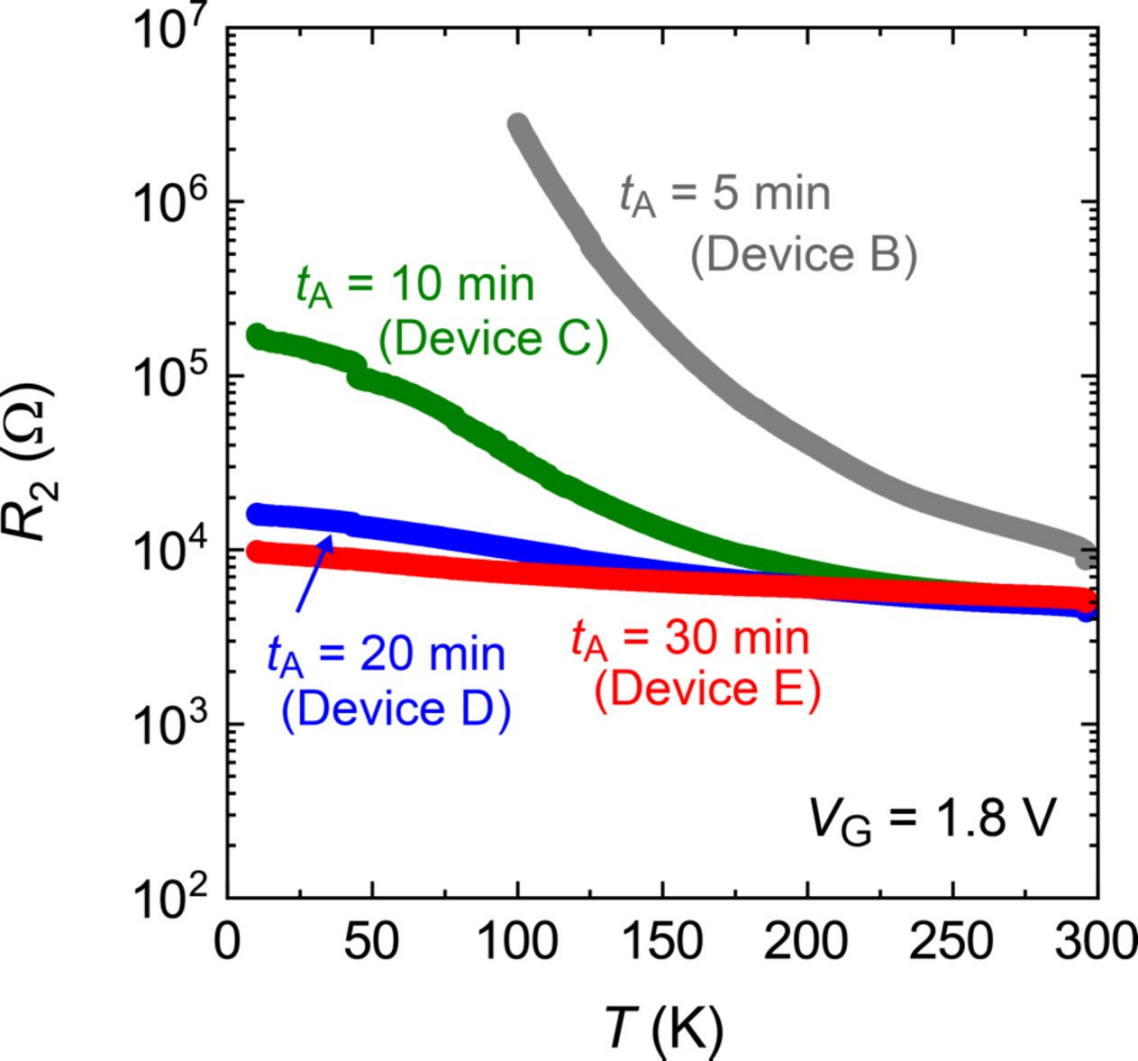
Comparison of temperature *T* dependence of two-terminal resistance *R*_2_ for different annealing times *t*_A_. The gate voltage *V*_G_ was maintained at 1.8 V for all the devices. With increasing *t*_A_ from 5 min for device B to 10 min for device C and 20 min for device D, *R*_2_ at low temperatures decreased. When *t*_A_ was further extended to 30 min for device E, the *T* dependence of *R*_2_ moderately changed, suggesting that the annealing effect was saturated at approximately *t*_A_ = 20 min.

At low temperatures, however, *R*_2_ exhibited a drastic contrast. To evaluate the *t*_A_ dependence of the contact resistance, we systematically measured *R*_2_ of the ion-gated InP with varying *V*_G_ and temperatures. Figure 2 shows the temperature dependence of *R*_2_ at *V*_G_ = 1.8 V for devices B, C, D, and E. When *t*_A_ = 5 min, *R*_2_ exhibited insulating behavior, even though the room-temperature value of *R*_2_ was suppressed to the order of 10 kW by gating. When we annealed the device for 10 min, *R*_2_ was measurable down to the lowest temperature of the present experiments, 10 K. For further expansion of *t*_A_, the low-temperature values of *R*_2_ were much more suppressed but did not show a drastic change when *t*_A_ was extended from 20 to 30 min. This suggests that the formation of the intermediate semiconductor layer at the electrode interface is almost saturated at around 20 min. See Supplementary Figure S1 for the temperature dependence of *R*_2_ on other *V*_G_ values.

**Fig. 3:**
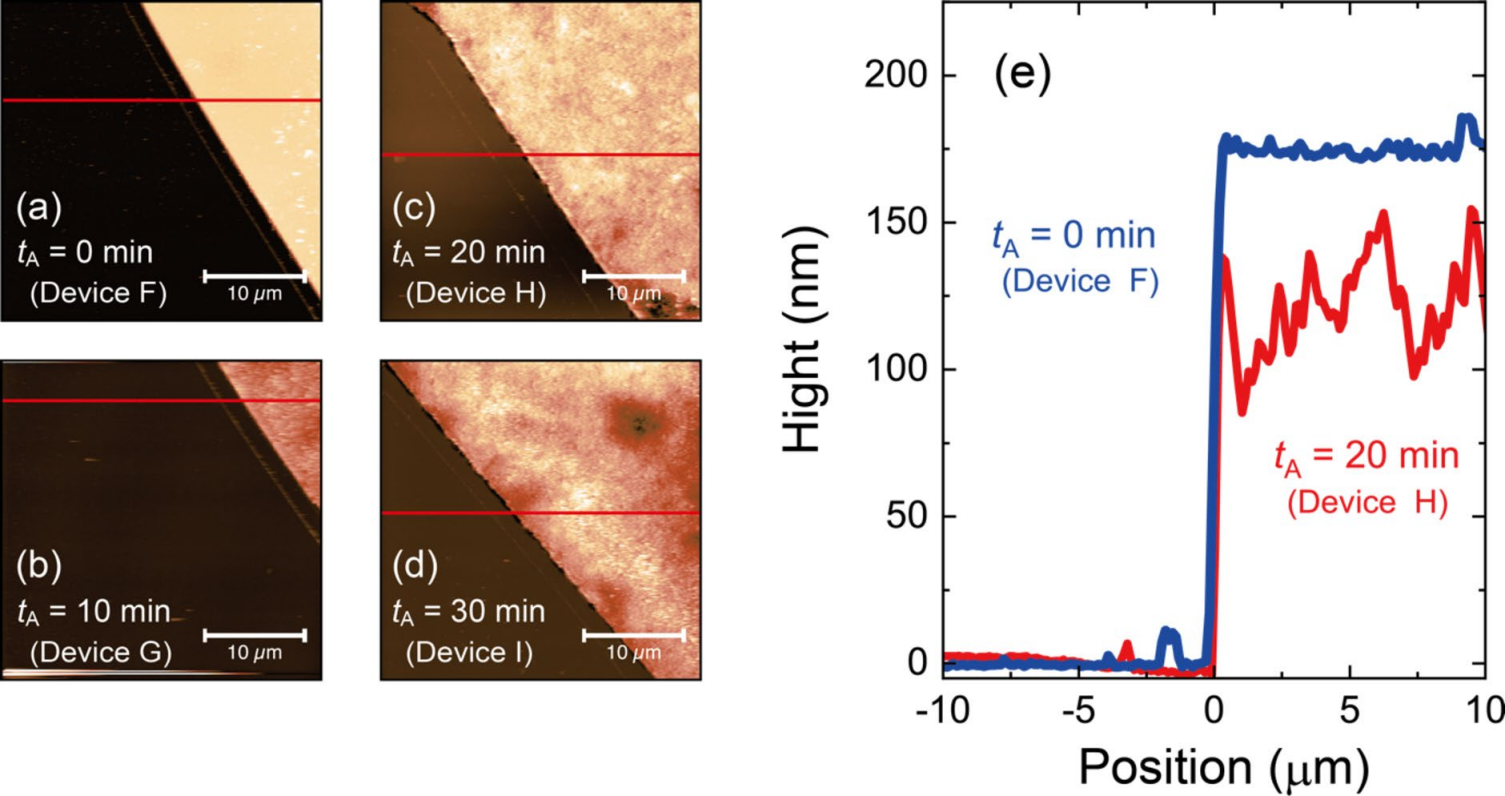
Atomic force microscopy (AFM) analyses of AuGe/Ni electrodes. (a)-(d) AFM topography image of boundary of InP and AuGe/Ni electrode. The four devices, F, G, H, and I, were prepared with the annealing times of *t*_A_ = 0, 10, 20, and 30 min, respectively. (e) Height profile along red lines in (a) and (c). The height of the AuGe/Ni electrode remained almost constant in device F with *t*_A_ = 0 min. The unevenness in the height of AuGe/Ni layers was confirmed in devices H, which was due to the atom migration through the high-temperature annealing process. See Supplementary Figure S2 for the height profiles of other devices.

The effect of annealing on the electrodes was also confirmed through the analysis with atomic force microscopy (AFM). For the AFM measurements, we prepared four devices: F (*t*_A_ = 0 min), G (*t*_A_ = 10 min), H (*t*_A_ = 20 min), and I (*t*_A_ = 30 min). Figure 3 shows the development of the surface morphology of the electrodes with increasing *t*_A_. At *t*_A_ = 0 min, the AFM topography image in Fig. 3a demonstrated a sharp boundary between the electrode and InP. With increasing *t*_A_ up to 30 min, we found that the surface and edges of the electrodes became uneven, as shown in Fig. 3b-d. We also investigated the height profile along the red lines in Fig. 3a-d and plotted them in Fig. 3e and Supplementary Fig. S2, where the origin of the x-axis was set at the edge of the electrodes. Figure 3e shows the data only for devices F and H for clarity. The height of the AuGe/Ni electrode was almost constant in device F with *t*_A_ = 0 min; in contrast, the heights of the AuGe/Ni layers exhibited unevenness in device H, which was more pronounced for devices with longer *t*_A_ (see Supplementary Fig. S2). This indicates that atom migration and the formation of the intermediate semiconductor layer continuously developed with *t*_A_ in this time scale. From the results in Figs. 2 and 3, we conclude that at least 20 min of annealing are required to obtain the low contact resistance that is valid for wide temperature regions. This value of *t*_A_ is much longer than those in previous studies, where the typical *t*_A_ ranged from several seconds to minutes^27–31^. Although the low temperature electrical transport measurement was enabled, the mechanism for reducing the contact resistance with long annealing time is unclear. Further investigations such as composition and structural analyses of the intermediate semiconductor layer is necessary, which will be conducted using a high energy X-ray bean in a synchrotron radiation facility and reported in a future work.

**Fig. 4:**
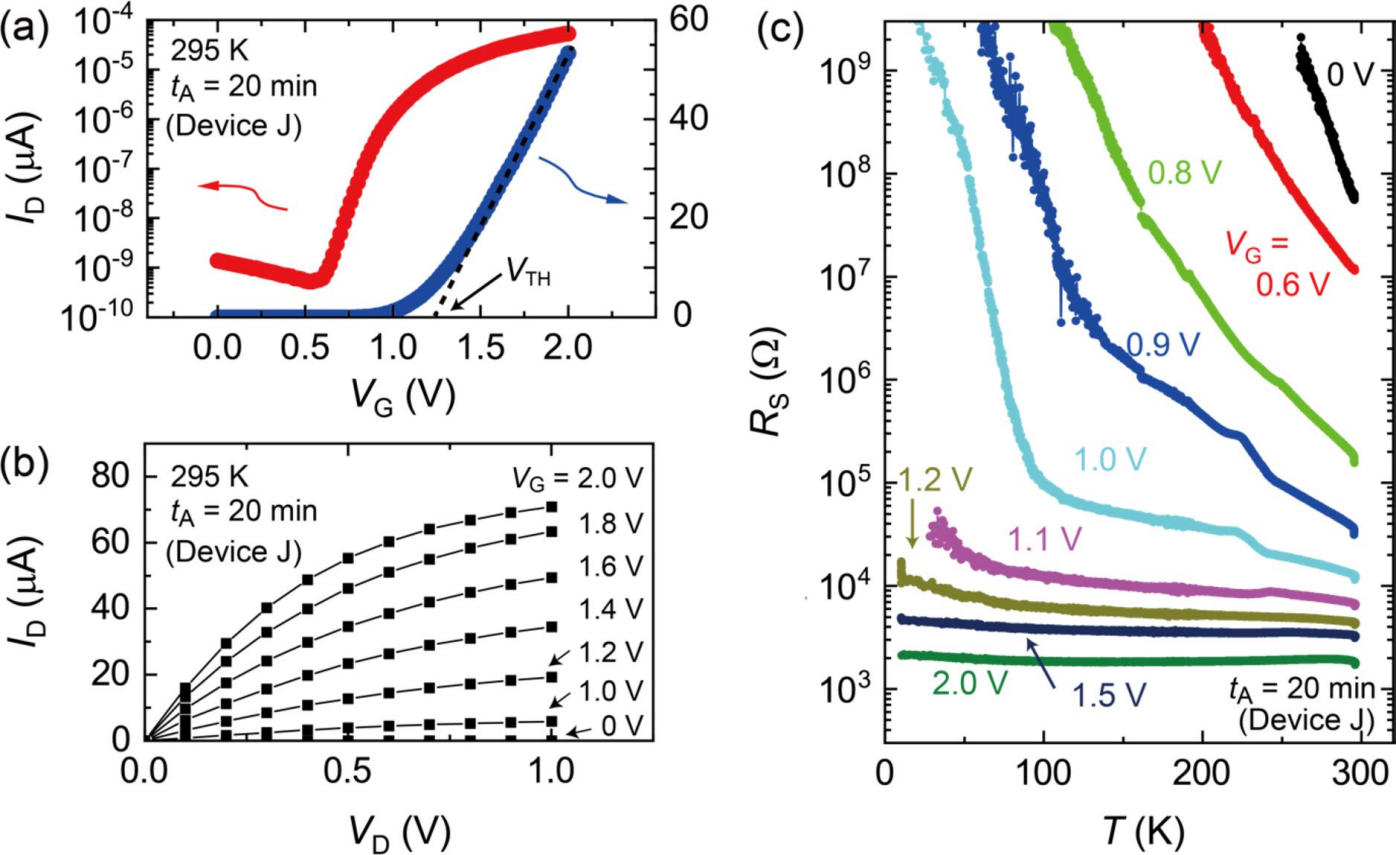
Insulator-to-metal transition in ion-gated InP. (a) Transfer curve (gate voltage *V*_G_ dependence of drain-source current *I*_D_) of device J at 295 K. The annealing time *t*_A_ of device J was 20 min. The drain-source voltage *V*_D_ was 0.5 V. The threshold voltage *V*_TH_ was estimated to be approximately 1.25 V from the slope of the curve. (b) Output characteristics (*V*_D_ dependence of *I*_D_) at 295 K of device J. A typical n-type field effect transistor operation was observed. (c) Temperature *T* dependence of sheet resistance *R*_S_ of device J. The values of *R*_S_ at low temperatures were suppressed with increasing *V*_G_, and an almost flat *T* dependences was observed when *V*_G_ was larger than 1.2 V.

After determining *t*_A_ = 20 min at 420 °C as an appropriate annealing condition, we fabricated another InP device with *t*_A_ = 20 min (device J) and systematically investigated the transistor characteristics. Figure 4a shows the transfer curves of device J at 295 K when the drain-source voltage *V*_D_ was 0.5 V. A negligibly small hysteresis was observed, where the forward and reverse scans overlapped. The threshold voltage *V*_TH_ was estimated to be approximately 1.25 V from the slope of the transfer curve, as indicated by the dashed line. As seen in the transfer curve plotted with a log scale, the subthreshold swing was found to be 93 mV/dec at *V*_G_ = 0.7 V, suggesting a potential for high-performance transistor operation. The ratio of the on- and off-currents was as large as 10^5^, indicating the large tunability of the carrier densities in the InP channel by the gate electric field. The FET properties were also confirmed by observing the output curves, as shown in Fig. 4b. Figure 4c shows the temperature dependence of the sheet channel resistance *R*_S_, which was estimated by normalizing the four-terminal resistance to the length and width of the channel (see Supplementary Fig. S3). The temperature dependence of *R*_S_ demonstrated insulating behavior when *V*_G_ was less than 1.2 V. With increasing *V*_G_, *R*_S_ was suppressed and finally showed a metallic behavior when *V*_G_ = 2.0 V was applied, achieving an electrically induced insulator-to-metal transition.

The insulator-to-metal transition shown in Fig. 4c is consistent with the transfer curve shown in Fig. 4a. As shown in Fig. 4c, the increase in *R*_S_ at low temperatures was suppressed by applying *V*_G_. The values of *R*_S_ showed divergence-like temperature dependencies below *V*_G_ = 1.2 V and shifted to nearly temperature independent behavior above *V*_G_ = 1.2 V. Meanwhile, in Fig. 4a, *V*_TH_ was about 1.25 V, above which *I*_D_ steeply increased in proportion to *V*_G_. Therefore, the insulator-to-metal transition in ion-gated InP would have occurred at *V*_G_ ≈ *V*_TH_. The reason that *R*_S_ versus temperature curves showed the insulating behaviors in the subthreshold regime, where *V*_G_ is lower than *V*_TH_, would be that the carrier density is not sufficient to maintain the electrical conduction at low temperatures^32–34^. In other words, percolative carrier doping is realized below *V*_TH_. It is suggested that the electrically induced carriers can be inhomogeneously doped because of the imperfect ordering of cations and anions^32,35^ and also the Coulomb potential induced by ions near the interface^36^, which makes mobile carriers localize at low temperatures. When *V*_G_ becomes larger than *V*_TH_, the chemical potential rises into the conduction band, and the electrons are accumulated according to the relationship between the sheet carrier density *N* and *V*_G_, *eN* = *C*_EDL_(*V*_G_-*V*_TH_). Here, *e* and *C*_EDL_ are the elementary charge and the capacitance of the electric double layer, respectively. Therefore, when a typical value of *C*_EDL_, 10 µF/cm^2 37–41^, is tentatively assumed, *N* at *V*_G_ = 2 V is 5 × 10^13^ cm^−2^. High-density electrons are induced in the FET channel in this *V*_G_ region; thus, the randomness of the carrier distribution is less dominant in the electrical conduction. Consequently, the gated surface electrons exhibited metallic conduction from room temperature to the lowest temperature as shown in Fig. 4c.

## Conclusions

We revealed the systematic evolution of the electrical transport properties of ion-gated InP against electrostatic carrier doping. With recent advancements in iontronics, it is highly required to expand the material variety of channels for ion-gated FETs. In particular, the application of III-V compound semiconductors to iontronic devices is promising because of their relatively high carrier mobilities and well characterized material properties. We fabricated the electric double layer transistors based on the semi-insulating InP single crystals. The n-type transistor operations were successfully observed by reducing the contact resistance at the electrode interface with AuGe/Ni Ohmic contacts through the annealing procedure. Ion gating is a versatile technique for carrier doping and continuous modulation of the carrier density at the surface of the InP single crystal, resulting in achieving the electrically-induced insulator-to-metal transition, which has been difficult to realize in ion-gated FETs based on bulk compound semiconductors. The present approach is applicable to other compound semiconductors, which would lead to the emergence of novel electronic properties and the development of the iontoronic devices.

## Methods

### Device fabrication

 To control the electron carrier density in InP, an electric double layer transistor structure was fabricated on the (111) surface of Fe-doped semi-insulating InP. An optical image of the device is presented in Supplementary Fig. S3. The single crystals of InP that we used in this study were doped with Fe to increase resistivity. We purchased the single crystals with electrical resistivity larger than 10^7^ Ωcm at room temperature from MO Sangyo Co., Ltd. The electrodes and channels were formed using standard photolithography and a metal evaporation process. We first evaporated the AuGe layer by resistance heating evaporation and then the Ni layer by electron beam evaporation, whose thicknesses were approximately 140 and 35 nm, respectively. To reduce the contact resistance, the devices were annealed after the lift-off process, as discussed in detail in the main text (see Supplementary Figure S4 for the comment on the contact resistance in Device J). A small amount of an ionic liquid, *N*,*N*-dimethyl-*N*-(2-methoxyethyl)-*N*-methylammonium bis-(trifluoromethylsulfonyl)-imide (DEME-TFSI), was deposited to cover both the channel and gate electrodes and perform gating experiments with a side gate configuration. When *V*_G_ is applied, an electric double layer is formed on the surface of the InP channel. The thickness of the electric double layer is order of 1 nm and works as a nano-gap capacitor in the transistor configuration. It has been reported that the capacitance of the electric double layer is as large as 10 µF/cm^2 37–41^ or larger. Here, it is noted that the strong electric field induced in the electric double layer can cause an unexpected chemical reaction on the channel surface. That is often accompanied by an irreversible modulation of electrical conductivity in channels. In Figs. 1c and 4a, the transfer curves were shown for both the forward and reverse scans. The reversibility of *I*_D_ and the negligible hysteresis suggest that the chemical reaction was unlikely to have occurred.

### Electrical measurements

 To evaluate the transfer characteristics (the gate voltage *V*_G_ dependence of the drain-source current *I*_D_) of the ion-gated InP, *I*_D_ was measured under the application of *V*_G_ and the drain-source voltage *V*_D_ with a semiconductor parameter analyzer (E5270, Agilent). The temperature dependence of the sheet resistance *R*_S_ was measured from 295 K to 10 K using a conventional Gifford-McMahon type refrigerator (PASCAL CO., LTD.) and a temperature controller (model 335, Lake Shore Cryotronics). In the ion-gated device structures, *I*_D_ can flow through the InP channel and the ionic liquid. See Supplementary Figure S5 for the evaluation of the resistance of the ionic liquid.

## Electronic supplementary material

Below is the link to the electronic supplementary material.

Supplementary Material 1

## Data Availability

The authors confirm that the data supporting the findings of this study are available within the article and the supplementary information.
