# Supplementary material for: Electrically induced insulator-to-metal transition in InP-based ion-gated transistor
Source: Sci Rep. 2024 Dec 5;14:30364. doi: 10.1038/s41598-024-81685-4 (PMC11621117; doi:10.1038/s41598-024-81685-4)
Supplement: Supplementary file 1 — Supplementary Material 1 [file 41598_2024_81685_MOESM1_ESM.pdf]

# Supplementary Information for "Electrically induced insulator-to-metal transition in InP-based ion-gated transistor"

Sunao Shimizu[1,*], Hiroki Shioya[2], Takafumi Hatano[3], Kazumoto Miwa[4], Akira Oiwa[5], and Shimpei Ono[6]

[1]Fucalty of Engineering, Toyama Prefectural University, Toyama, 939-0398, Japan

[2]R[3] Institute for Newly-Emerging Science Design, Osaka University, Osaka, 560-8531, Japan

[3]Department of Materials Physics, Nagoya University, Nagoya, 464-8603, Japan

[4]Materials Science Division, Central Research Institute of Electric Power Industry (CRIEPI), Kanagawa, 240-0196, Japan

[5]SANKEN, Osaka University, Osaka, 567-0047, Japan

[6]International Center for Synchrotron Radiation Innovation Smart (SRIS), Tohoku University, Miyagi 980-8572, Japan

[*]s-shimizu@pu-toyama.ac.jp

## Supplementary Figures

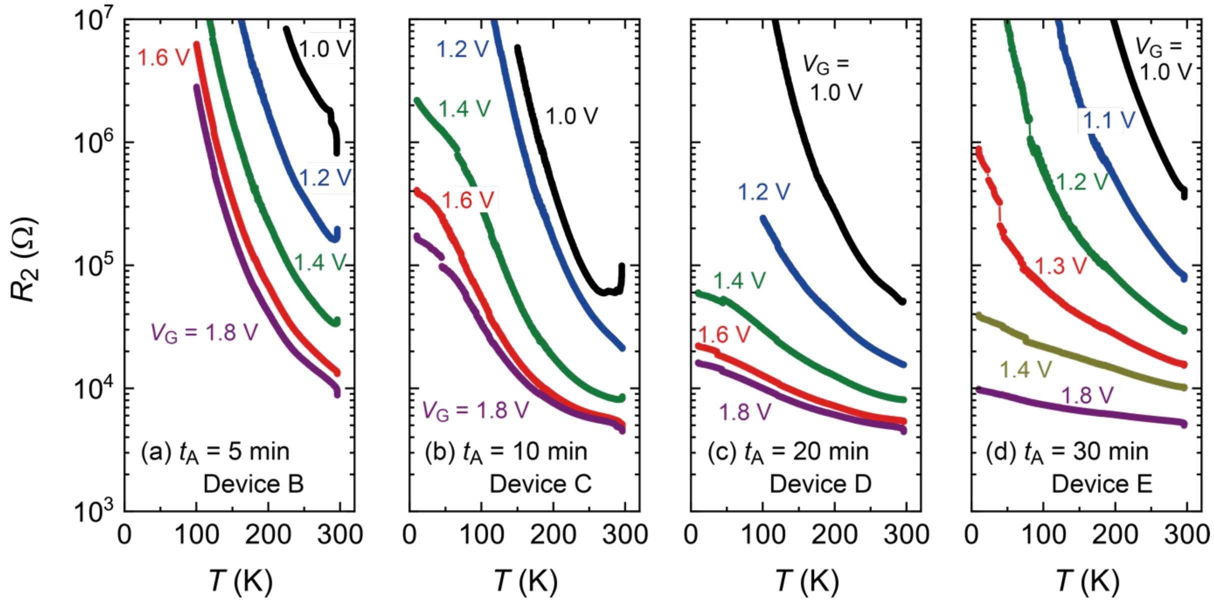


**Supplementary Figure S1**. Temperature $T$ dependence of two-terminal resistance $R_2$ of ion-gated InP. When the annealing time $t_A$ was 5 minutes, $R_2$ showed an upturn with decreasing temperature for all the gate voltage $V_G$, as shown in (a). The valued of $R_2$ became too high, which made it difficult to measure $R_2$ in the low $T$ region below 100 K. When we annealed the sample for 10 minutes, $R_2$ were measurable down to 10 K, as shown in (b), when $V_G$ was larger than 1.4 V. For further expansion of $t_A$, the low $T$ values of $R_2$ were much more suppressed as shown in (c) and (d).

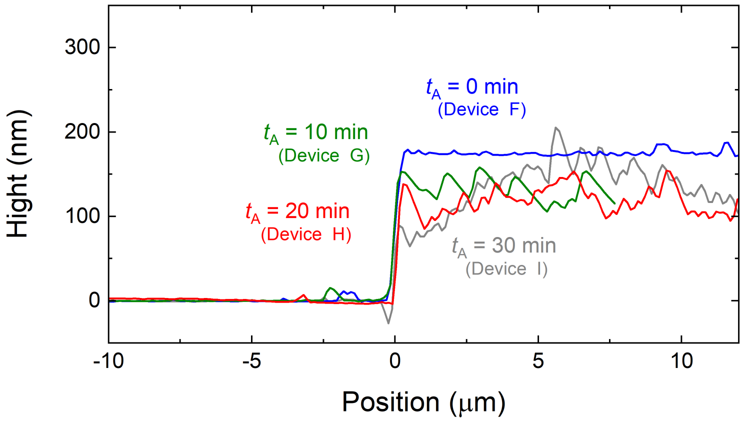


**Supplementary Figure S2.** Hight profiles of AuGe/Ni electrodes on InP with atomic force microscopy (AFM). For the AFM measurements, we prepared four devices: F ($t_A$ = 0 min), G ($t_A$ = 10 min), H ($t_A$ = 20 min), and I ($t_A$ = 30 min), where $t_A$ is annealing time. The height profiles were investigated along the red lines in Fig. 3a-d in the main text. The origin of the x-axis was set at the edge of the electrodes. The height of the AuGe/Ni electrode exhibited unevenness in the annealed devices, which was more pronounced for devices with longer $t_A$.

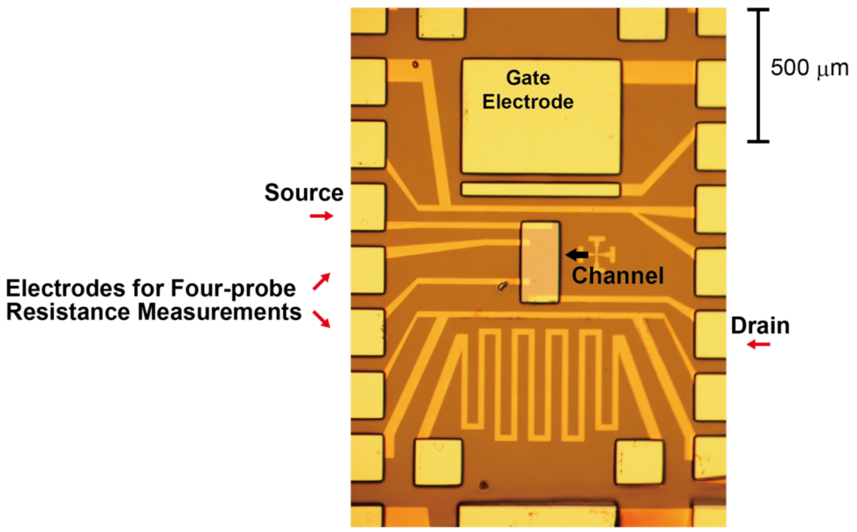


**Supplementary Figure S3.** Optical image of ion-gated InP device. The electrodes were patterned using a standard photolithography technique. The device was covered by hard baked photoresist except for the channel, the gate electrode, and the contact pads. The length and the width of the channel was 300 μm and 150 μm, respectively. The ionic liquid and the metal wires were removed to clearly demonstrate the device structure. See "Experimental Section" in the main text for the device fabrication process.

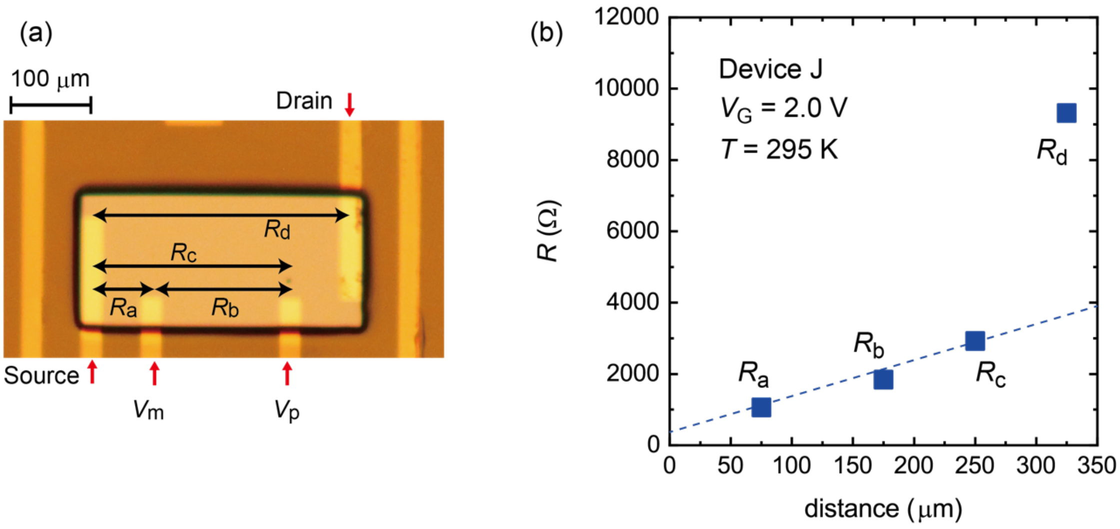


**Supplementary Figure S4** Evaluation of Resistance against distance between electrodes. (a) Optical image of channel area of ion-gated InP device. We define $R_a$, $R_b$, $R_c$, and $R_d$ as the two-terminal resistance for the areas assigned by the arrows. (b) Distance dependence of resistance for Device J at $V_G$ = 2.0 V and $T$ = 295 K. The dashed line is the guide to the eye. The resistance increased with increasing the distance; however, $R_d$ departed from the dashed line. This suggests that contact resistance under the drain electrode was much larger than those for other three electrodes in Device J and that the contact resistances in other devices used in the present study would not be uniform as well. Further investigation on the annealing condition is required to realize a low and uniform contact resistance.

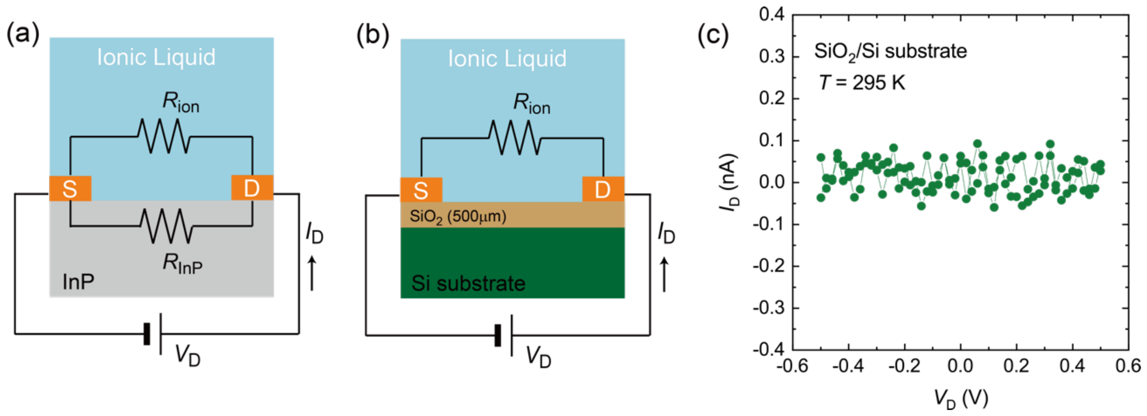


**Supplementary Figure S5** When a voltage $V_D$ is applied between the drain (D) and the source (S) electrodes in the ion-gated InP, an electric current $I_D$ can flow in InP and the ionic liquid. This situation is assumed to be the parallel conduction for InP and ionic liquid, as schematically shown in (a). Therefore, the resistance measured in experiments can be the combined resistance of $R_{ion}$ and $R_{InP}$, where $R_{ion}$ and $R_{InP}$ are the resistance of ionic liquid and the InP channel, respectively. For the estimation of $R_{ion}$, we fabricated the same patterns of electrodes on a $SiO_2$/Si substrate with that of the ion-gated InP devices. The $SiO_2$ layer is completely insulating, so that only $R_{ion}$ is considered, as schematically shown in (b). The $V_D$ dependence of $I_D$ in the configuration in (b) is shown in (c). The value of $I_D$ was lower than the noise level of our experimental setup. If we assume that the upper limit of $I_D$ is ~0.05 nA at $V_D$ = 0.5 V, $R_{ion}$ would be order or $10^{11}$ Ω or larger.
